# Sarcoidosis and Histoplasmosis: Is One a Consequence of the Other? A Case Report and Review of the Literature

**DOI:** 10.1155/2015/108459

**Published:** 2015-08-06

**Authors:** Anupam Bansal, Rupali Drewek

**Affiliations:** ^1^Atlantic University School of Medicine, Gros Islet Highway, Rodney Bay, Saint Lucia; ^2^Phoenix Children's Hospital, 1919 E. Thomas Road, Phoenix, AZ 85016, USA

## Abstract

Sarcoidosis involves abnormal collections of inflammatory cells (granulomas) which may form as nodules in multiple organs. 90% of affected patients have respiratory tract abnormalities. We present a 61-year-old male with sarcoidosis who was admitted for respiratory distress. Fibrosing mediastinitis was seen in the chest computograph. Management was conservative and included steroids, antibiotics, and oxygen therapy. Sarcoidosis and fibrosing mediastinitis are rare. Fibrosing mediastinitis is more commonly seen with histoplasmosis. We explore the clinical similarities between histoplasmosis and sarcoidosis. We also explore the potential cause and effect relationship and workup for each disease entity.

## 1. Introduction

Sarcoidosis involves abnormal collections of inflammatory cells (granulomas), which may form as nodules in multiple organs. It is considered a diagnosis of exclusion and most commonly presents with respiratory symptoms. Histoplasmosis is caused by the fungus* H. capsulatum* and causes symptoms that are almost identical to those present with sarcoidosis. Clinicians rely on patient history, lab values, and radiological and pathological studies to distinguish between them. However, there are false negative tests and atypical findings which may complicate the diagnosis. Misdiagnosing one for the other and starting the incorrect treatment plan could cause significant morbidity. In this case, we present a male patient diagnosed with sarcoidosis. History was obtained by personal interview and clinic and hospital medical records. His radiographic finding of fibrosing mediastinitis is rare in sarcoidosis and more commonly seen with histoplasmosis. We review this case to make clinicians aware of the striking resemblance between these two entities and the workup that is recommended to accurately diagnose each disease.

## 2. Case Presentation

**Figure 1:**
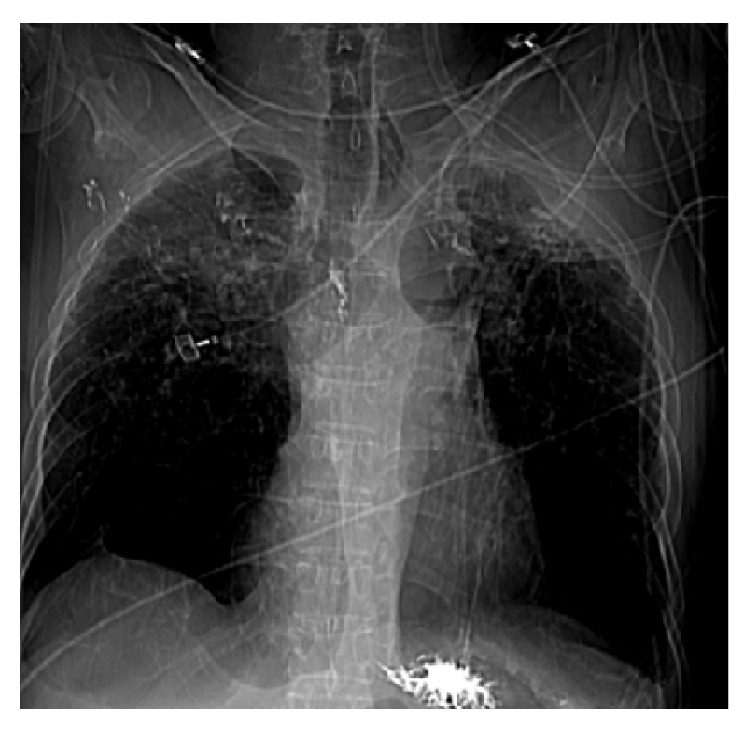
Chest X-ray (CXR) showed extensive fibrocalcific scarring in the upper lobes and compensatory emphysema of the lower lobes.

A 61-year-old male with past medical history significant for sarcoidosis was admitted with acute respiratory distress. He had severe baseline lung disease with the most recent forced expiratory volume in 1 second (FEV1) at 18%. Arterial blood gas analysis revealed respiratory acidosis with a pH of 7.27 and CO_2_ of 57 (on 50% FiO_2_). Due to his level of respiratory distress, he was intubated with ventilator support. He was treated with antibiotics, steroids, and airway clearance measures. Chest X-ray (CXR) showed extensive fibrocalcific scarring in the upper lobes and compensatory emphysema of the lower lobes (Figure 1). Cultures from the endotracheal tube were negative. Viral Direct Fluorescence Antibody (Viral DFA) was negative. Echocardiogram (ECHO) showed normal left ventricular systolic function with an ejection fraction of 60%, right ventricular enlargement, diastolic dysfunction, and normal pulmonary artery pressures. Ventilation/Perfusion Scan (V/Q Scan) was negative for pulmonary embolism.

Our patient first presented with respiratory symptoms at 28 years of age. His symptoms at that time included intermittent fatigue, shortness of breath, a “sore” tongue, and “facial swelling.” While spirometry was normal, his CXR revealed left hilar adenopathy and calcification. Mediastinoscopy was performed, including a mediastinal lymph node biopsy where he was found to have noncaseating granulomas. The fungal cultures and AFB (acid fast bacilli) stains by bronchoscopy were negative. The patient was diagnosed with sarcoidosis and started on prednisone for several months, to which his symptoms responded favorably. For 2 decades, he was treated with inhaled steroids and intermittent courses of prednisone. In his 40s, he presented to an ENT (ear, nose, and throat) specialist with uveitis and a hoarse voice. The ENT physician felt that the cause of the hoarse voice was iatrogenic (secondary to inhaled steroids). No biopsy was performed. At the age of 55, a cavitary lesion was detected on chest CT, and sputum cultures were positive for* Aspergillus*. For this reason, oral antifungal therapy was instituted.

**Figure 2:**
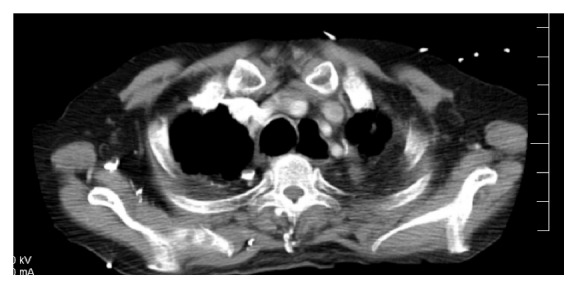
Chest computography shows the dilated esophagus above the fibrosing mediastinum.

**Figure 3:**
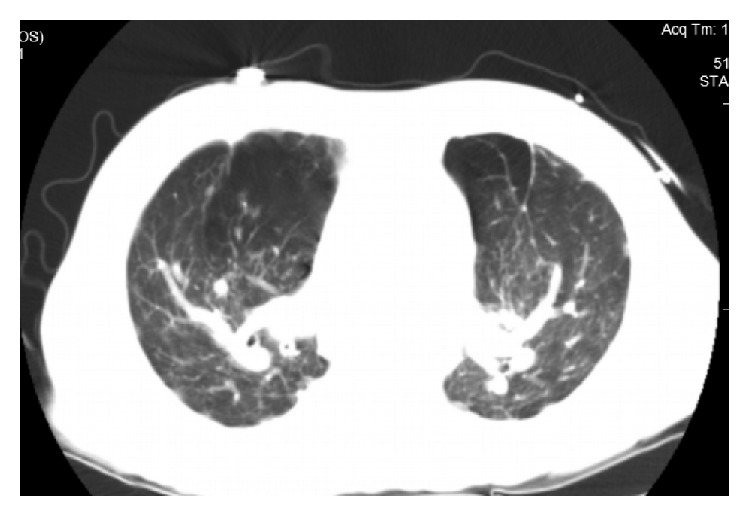
Chest computography with extensive calcification, scarring, and areas of hyperinflation.

Initial CXRs at the age of 28 showed left hilar and left paratracheal adenopathy. There were also calcified nodules in right and left lung fields. CXRs from the age of 45 through 52 showed progressive upper lobe fibrotic changes. From the age of 52 to 62, the images were stable. The CT scan during the most recent admission was stable as compared to the one done 3 years prior. It showed extensive calcification and soft tissue within the mediastinum with retraction of the mediastinum consistent with fibrosing mediastinitis. Both upper lobes had extensive calcifications and scarring. The esophagus was dilated above the fibrosing mediastinum (Figures 2 and 3). Pulmonary lung function just before admission showed an FVC of 56% FEV1 22% with a ratio of 35%. There was no postbronchodilator response. DLCO (diffusion capacity) was 69%. TLC was 62%, FRC was 75%, and RV was 69%. These values were unchanged as compared to 5 years ago. MRI of the brain showed no evidence of neurosarcoidosis.

Socially, the patient was a nonsmoker and was not exposed to any known environmental toxins which could cause interstitial lung disease. His immunizations were up to date including BCG. He was born in India and lived in Ohio at the age of 20–30 years and in Illinois thereafter. There was no known family history of lung disease.

## 3. Discussion

Sarcoidosis and histoplasmosis have a striking resemblance in terms of clinical presentation.  Table 1 outlines the shared clinical characteristics [1, 2]. The radiographic finding of fibrosing mediastinitis is commonly associated with histoplasmosis [3]. The fact that our patient lives in Ohio (where histoplasmosis is most common) brings up the question of whether our patient could have had a concurrent histoplasmosis infection at the time of initial presentation. This scenario would complicate management since treatment with immunosuppressants for sarcoidosis would worsen any underlying infection. Even though bronchoscopy cultures were negative for fungal infections, there is a significant rate of false negatives [4]. Wheat et al. described 11 patients diagnosed as having sarcoidosis who had laboratory evidence of histoplasmosis [4]. The diagnosis of histoplasmosis in these patients was confirmed by urine antigen, serologic tests, and/or immunodiffusion. These patients all received steroids prior to the diagnosis of histoplasmosis being confirmed. Therefore, it is difficult to say whether histoplasmosis was the primary diagnosis or was a secondary diagnosis secondary to an immunocompromised state. Baughman described 2 cases where sarcoid patients with declining respiratory status attributed their symptoms to sarcoidosis [5]. Their corticosteroid dosage was increased and their condition deteriorated even further. Ultimately, they were detected to have culture evidence of histoplasmosis. Reimann described a case of histoplasmosis in Pennsylvania which was mistakenly diagnosed as sarcoidosis [6]. Sharma described cases where diffuse histoplasmosis was found in a patient with sarcoidosis [7]. It is evident that misdiagnosis can happen and an incorrect treatment plan can cause significant morbidity.

Sarcoidosis is a disease process with unknown etiology. Various genetic, environmental, and infectious processes have been hypothesized.* Mycobacterium* has been the longest hypothesized and most investigated potential etiology of sarcoidosis [8]. Other infectious etiologies proposed include* Propionibacterium acnes* and certain viruses [9]. Mycetic agents have also been proposed as potential etiologies [9]. We explored the possibility of histoplasmosis as a causal agent for development of sarcoidosis. Israel et al. investigated a case of chronic disseminated histoplasmosis and its relationship to sarcoidosis [10]. He suggested that histoplasmosis could be the primary disease and stimulate the sarcoidosis [10]. Wynbrandt and Crouser described a case in which documented pulmonary histoplasmosis allegedly evolved into sarcoidosis [11]. Interestingly, there seems to be a higher prevalence of sarcoidosis in Franklin County, Ohio (the demographic profile of which is nearly identical to that of the US) [12]. Ohio is the state where histoplasmosis is also most commonly found.

In addition to similar clinical findings, both histoplasmosis and sarcoidosis present with similar radiographic findings. Chest X-ray will show hilar lymphadenopathy, reticulonodular opacities, nodules, and air trapping. Fibrosing mediastinitis is more commonly found with histoplasmosis. However, it is (albeit rarely) associated with sarcoidosis. Devaraj et al. reported two cases in a series of 12 patients with CT images and histology of fibrosing mediastinitis (16% of patients) [13]. There were 3 separate case reports discussing fibrosing mediastinitis in patients with sarcoidosis developing complications such as pulmonary edema, pulmonary hypertension, and pulmonary artery occlusion [14–16]. Strock recently found that certain HLA typing within histoplasmosis could carry a higher predisposition towards development of fibrosing mediastinitis [17]. This suggests that an aberrant host immune response could be contributing to its pathogenesis.

There are other clinical markers which can be used to diagnose sarcoidosis. However, these also can be misleading. ACE (angiotensin converting enzyme) levels are usually only elevated in sarcoidosis but there was a reported case where a patient had elevated ACE levels and* Histoplasma* grew from the liver biopsy specimens [7]. ACE levels can also have false positive and negative rates [18]. Noncaseating granulomas, which are the hallmark for sarcoidosis, are also seen in tissues from patients with histoplasmosis [19]. Histoplasmosis usually exhibits few angular ragged granulomas, while sarcoidosis usually contains more granulomas and has a rounded morphology [20]. A positive Kveim-Siltzbach test provides strong support for the diagnosis of sarcoidosis [21]. However, this test is rarely performed.

Laboratory methods to diagnose histoplasmosis include cultures, histology, molecular techniques, serology, and antigen detection.* Histoplasma* organisms are difficult to visualize in tissues and have often been initially overlooked [22]. In addition, the sensitivity can be low (9–34%) especially in acute or localized cases [23, 24]. Histopathological evaluation oftentimes lacks sensitivity. Urine antigen sensitivity was noted to be 93–100% [25]. In recent years,* Histoplasma capsulatum* has been identified in clinical samples by a highly sensitive molecular marker, a fragment of the Hcp 100 gene. PCR assays for the Hcp 100 gene have been validated in several studies and have shown high specificities [26–29].

In summary, chronic disseminated histoplasmosis and sarcoidosis have strikingly similar features. Diagnostic tests including radiography also have similarities. Unfortunately, applying loose criteria for the diagnosis of sarcoidosis may result in overlooking specific infectious diseases including histoplasmosis. Cultures alone cannot exclude histoplasmosis due to a significant rate of false negatives. As a result, the recommended workup to accurately differentiate between sarcoidosis and histoplasmosis should include radiography, tissue biopsies, cultures, ACE level, serology, PCR assay, and urine antigen. Future studies will be needed to investigate the link between these two diseases.

## Figures and Tables

**Table 1:** Shared characteristics and differences between histoplasmosis and sarcoidosis.

	Histoplasmosis	Sarcoidosis
Clinical symptoms	Cough	Cough
	Dyspnea	Dyspnea
	Adrenal involvement	Adrenal involvement
	Erythema nodosum	Erythema nodosum
	Splenomegaly	Splenomegaly
	Anorexia	Anorexia
	Fever	Fever
	Arthralgia	Arthralgia
	Skin ulcers	Skin ulcers
	Uveitis/retinitis	Uveitis/retinitis
	Hepatitis	Hepatitis
	Parotid gland involvement	Parotid gland involvement
	Cervical myelopathy	Cervical myelopathy

Laboratory findings	Histology	Histology: noncaseating granulomas
	Cultures (bronchoalveolar lavage)	Elevated angiotensin converting enzyme
	Immunoassays	Elevated vitamin D
		Gammaglobulinemia
		Kveim

Radiographic findings	Hilar lymphadenopathy	Hilar lymphadenopathy
	Fibrosing mediastinitis	Upper lobe involvement
	Nodules	Nodules
	Reticulonodular opacities	Reticulonodular opacities
		Granulomas along lymphatic vessels
		Air trapping

Treatment	Itraconazole/amphotericin	Prednisone
	Streptomycin	
	Bacillomycin B	
